# When a Friend Becomes Your Enemy: Natural Killer Cells in Atherosclerosis and Atherosclerosis-Associated Risk Factors

**DOI:** 10.3389/fimmu.2021.798155

**Published:** 2022-01-13

**Authors:** Maria Teresa Palano, Martina Cucchiara, Matteo Gallazzi, Federica Riccio, Lorenzo Mortara, Gian Franco Gensini, Gaia Spinetti, Giuseppe Ambrosio, Antonino Bruno

**Affiliations:** ^1^ Laboratory of Innate Immunity, Unit of Molecular Pathology, Biochemistry and Immunology, Istituto di Ricovero e Cura a Carattere Scientifico (IRCCS) MultiMedica, Milano, Italy; ^2^ Laboratory of Immunology and General Pathology, Department of Biotechnology and Life Sciences, University of Insubria, Varese, Italy; ^3^ Laboratory of Cardiovascular Physiopathology-Regenerative Medicine, Istituto di Ricovero e Cura a Carattere Scientifico (IRCCS) MultiMedica, Milano, Italy; ^4^ Istituto di Ricovero e Cura a Carattere Scientifico (IRCCS) MultiMedica, Milano, Italy; ^5^ Division of Cardiology, University of Perugia, Perugia, Italy

**Keywords:** natural killer cells, atherosclerosis, atherosclerosis-related risk factors, type-2 diabetes, obesity

## Abstract

Atherosclerosis (ATS), the change in structure and function of arteries with associated lesion formation and altered blood flow, is the leading cause of cardiovascular disease, the number one killer worldwide. Beyond dyslipidemia, chronic inflammation, together with aberrant phenotype and function of cells of both the innate and adaptive immune system, are now recognized as relevant contributors to atherosclerosis onset and progression. While the role of macrophages and T cells in atherosclerosis has been addressed in several studies, Natural Killer cells (NKs) represent a poorly explored immune cell type, that deserves attention, due to NKs’ emerging contribution to vascular homeostasis. Furthermore, the possibility to re-polarize the immune system has emerged as a relevant tool to design new therapies, with some succesfull exmples in the field of cancer immunotherapy. Thus, a deeper knowledge of NK cell pathophysiology in the context of atherosclerosis and atherosclerosis-associated risk factors could help developing new preventive and treatment strategies, and decipher the complex scenario/history from “the risk factors for atherosclerosis” Here, we review the current knowledge about NK cell phenotype and activities in atherosclerosis and selected atherosclerosis risk factors, namely type-2 diabetes and obesity, and discuss the related NK-cell oriented environmental signals.

## Introduction

Cardiovascular diseases (CVDs), the first cause of death worldwide, are characterized by an inflammatory microenvironment (1–3). An altered immune response can govern and impact both pathological resolution and/or progression of CVDs, as a consequence of systemic and tissue-local environments (4–7). In this context, it is worth noticing that atherosclerosis (ATS), the leading cause of CVDs (8), is characterized by lipids accumulation, cell apoptosis, endothelial cells (ECs) increased permeability, fibrosis, and chronic inflammatory burden (9). In particular, EC dysfunction in ATS leads to proinflammatory cytokines production and immune cells recruitment within the atherosclerotic plaque (9). This tight connection between inflammation and CVDs has been recently reinforced by the results of two clinical trials (Canakinumab Anti-inflammatory Thrombosis Outcomes Study-CANTOS, and Low Dose Colchicine for Secondary Prevention of Cardiovascular Disease-LoDoCo) that show the benefit of targeting inflammation to lower the risk of CV events (10, 11). CANTOS investigators found that canakinumab (an interleukin-1β, IL-1β, neutralizing antibody) exerts a protective effect on thrombosis, targeting the IL-1β innate immunity pathway. Interestingly, the study shows that compared to placebo, the treatment also correlates with lower cancer mortality. Furthermore, the LoDoCo trial showed a preventive role of colchicine in the occurrence of cardiovascular events with inhibition of neutrophil chemotaxis and activation within a proinflammatory environment. In addition, another study showed that heart failure–associated inflammatory markers, including C‐reactive protein, at the same time had a clear predictive value of new-onset cancer, independently of cancer risk factors (12). These data suggest that cancers and CVDs share some common mechanisms centered on inflammation and the immune response that may represent valuable novel targets for therapies. Moreover, ATS and cancer share risk factors such as obesity, diabetes mellitus, and hypertension (13), and pathophysiological pathways, such as chronic inflammation, oxidative stress, and alterations in immune cells phenotype and functions. Of note, immune cells, both of innate and adaptive immunity, are characterized by extraordinary plasticity, thus they can adapt their phenotype and response (referred as immune cell polarization) to the hosting pathophysiological micro-(tissue/local) and macro-(peripheral blood/systemic) environment (14–19). These adaptation capabilities result in the ability of immune cells to acquire contrasting activities, as related to their “original commitment”, which is the defense of the host organism. These peculiar features have been observed in diverse chronic inflammatory-based disorders, ranging from CVD (6, 20, 21), to autoimmune diseases (22–24), to cancers (25–27).

The most investigated players in ATS are monocytes/macrophages, which, upon activation, support the subsequent specific T and B cells response (28, 29). However, recent preclinical and clinical evidence point to a role in ATS also for NK cells (30, 31), which are large granular lymphocytes of innate immunity, primarily involved in the immunosurveillance against virus-infected and malignant-transformed cells. NK cells can infiltrate the vessel wall, promoting atherosclerotic lesion development and producing perforin and granzyme B, thus leading to more vulnerable atherosclerotic lesions for atherothrombosis (32, 33).

Here, we review the current knowledge of NK cell phenotype and activities, discussing the environmental cues that can instruct NK cell behavior in ATS pathological context.

### NK Cells

NK cells originate in the bone marrow from CD34^+^ hematopoietic stem cell (HSC) precursors, which generate common lymphoid progenitors (CLP), further committed to the NK cell progenitor (NKP), following the acquisition of IL-2/IL-15Rβ subunit (CD122) that makes NK cells responsive to IL-15. Mature peripheral blood NK cells express CD16, CD57, and NKG2D, and display the ability to release perforin, granzyme, and IFNγ (34–36).

The Neural Cell Adhesion Molecule/NCAM (CD56) and the FC gamma receptor III molecule (CD16) are the two major surface antigens used to discriminate NK cell subsets. CD56^+^CD16^+^ NK cells (90-95% of total circulating NKs) are endowed with cytolytic functions, *via* antibody-dependent cellular cytotoxicity (ADCC), and release of perforin and granzymes, thus mediating the immunological synapsis between target and effector cell complex (34, 37). CD56^bright^CD16^-^, (5-10% of circulating NKs) (34, 37) act by releasing pro-inflammatory and anti-tumor cytokines, such as IFNγ, TNFα. Several studies, in particular within the recent single-cell era, demonstrated that NK cell subset classification may extend beyond this classical dichotomy of CD56^dim^CD16^+^ and CD56^bright^CD16^-^ NKs (38–41).

The local microenvironment, with its unique cellular interactions, provide relevant signals to shape NK cell phenotype and functions, both under physiological and pathological conditions (18, 42–45). During fetal development, NK cells acquire a peculiar phenotype, described as CD56^superbright^CD16^-^, termed decidual NKs (dNKs), endowed with increased ability to produce pro-angiogenic factors, such as vascular endothelial growth factor (VEGF), placental growth factor (PlGF), and CXCL8 (46, 47). Thus, in this particular scenario, dNK cells shift from killer to builder effector cells, being necessary for the appropriate formation of spiral arteries, which deliver oxygen and nutrients to the developing fetus (46, 47). Intriguingly, an expansion of pro-angiogenic, decidual-like NK cell subset has been found in solid cancers: these dNK-like, termed tumor-infiltrating (those present in tumor tissues-TINKs) and tumor-associated (NKs in the peripheral blood-TANKs) NK cells acquire a CD56^bright^CD16^low^CD9^+^CD49a^+^ phenotype, can release pro-angiogenic factors and support EC proliferation, migration and ability to form capillary-like structures (18, 45, 48–51). Major mechanisms governing the alterations of NK cell activity in chronic inflammatory disorders include downregulation of the activation molecule NKG2D and the Natural Cytotoxicity Receptors (NCRs) NKp46, NKp44, and NKp30, together with decreased or increased capability to release IFNγ, TNFα, perforin, and granzymes, according to the hosting environment (52–54).

### NK Cells in Atherosclerosis and Atherosclerosis-Related Risk Factors

ATS represents the most common pathophysiological alteration leading to ischemic heart disease and stroke (55). NK cells have been detected in atherosclerotic lesions in humans (56, 57) and mice (32, 33, 58, 59), mostly in advanced lesions, deep within plaques, and in shoulder regions of plaque (32). In addition, patients with advanced lesions show high levels of circulating NK cells (60). Here, we focus on NK in ATS and selected ATS-associated risk factors, i.e. type-2 diabetes, and obesity, analyzing the inflammatory environmental factors and signaling orchestrating different NK cells phenotype and functions, together with their interactions with other cells of the host organism.

### NK Cells in Atherosclerosis

There is contrasting evidence on the direct contribution and regulatory function of NK cells in ATS insurgence and progression, mainly as a consequence of the different murine models employed in various *in vivo* studies.

The first experiments investigating the role of NK cells in ATS have been conducted in beige mice, an animal model of NK cell functional deficient. In this murine model, Paigen et al. (61) reported no difference in ATS lesion size, suggesting no crucial role for NK cells in ATS. A second study using the NK cell functional deficient beige mice, crossed with LDLR deficient mice (beige, *LDLr*-/-), showed increased lesion size, as compared to control *LDLr*-/- mice, fed a high-fat diet (HFD). When reconstituted with the bone marrow (BM) of Ly49A transgenic mice (a murine model overexpressing the Ly49A receptor under the control of granzyme A promoter), *LDLr^-/-^* recipient animals exhibited smaller sizes of the lesions (62).

Further studies performed in *ApoE* ^-/-^ mice, in which NK cells were depleted by anti-asialo-GM-1 antibody, showed a significant reduction of the atherosclerotic lesion development (63). However, since several other cell types, such as myeloid cells, epithelial cells, and T-cell subsets express the glycolipid asialo-GM1 (64–67), the effects observed cannot be considered restricted to NK cells.

In another study, NK cells pre-activated with IL-2 were adoptively transferred into *ApoE^−/−^ Rag2^−/−^ IL2rg^−/−^* mice, resulting in increased ATS and necrotic core development, and IFNγ, Perforin, and Granzyme B production, by transferred NK cells (32).

**Figure 1:**
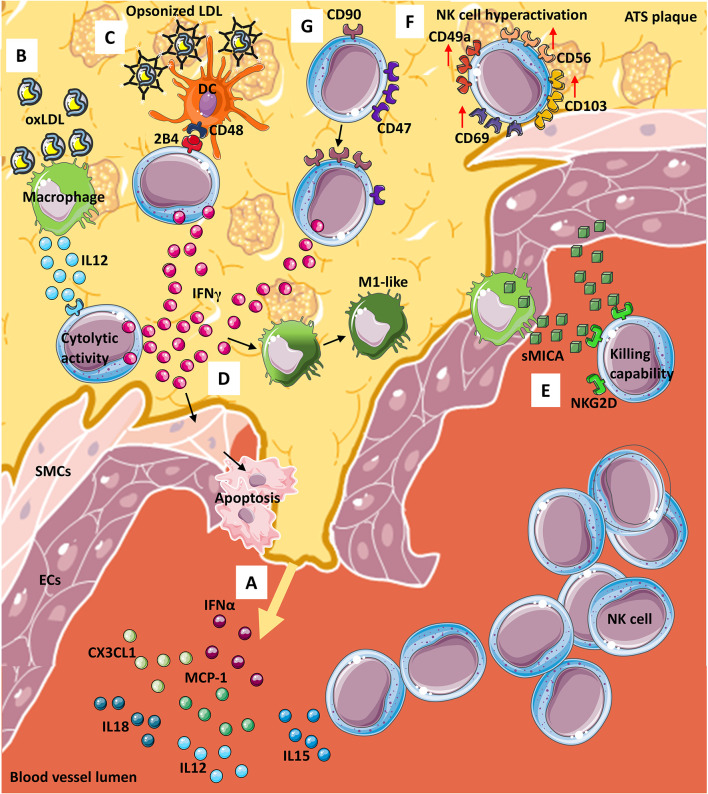
NK cells in atherosclerosis. From the site of atherosclerotic (ATS) plaque formation **(A)** many cytokines and chemokines are released into the blood, stimulating NK cells migration and entry within the plaque. Infiltrating NK cells can interact, by contact, with other immune cells such as **(B)** macrophages, that in presence of oxLDL, produce and release IL-12, thus potentiating cytolytic activity of NK *via* interferon γ (IFNγ) release. OxLDL can also be present as opsonized particles **(C)** that support dendritic cells (DCs)-NK crosstalk which is also mediated by CD48-2BA interaction and leads to **(D)** IFNγ release from NKs. IFNγ from NK cells exerts its effect on both **(D)** smooth muscle cells (SMCs) by inducing their apoptosis and macrophages by promoting M1-like phenotype switch. Furthermore, both **(E)** macrophages and endothelial cells (ECs) release soluble MICA (sMICA) that upon NKG2D binding on NK cells increase NK killing capability. Within ATS plaque, **(F)** a subset of NK cells display a hyperactive phenotype/behavior with increased expression of CD49a, CD56, CD69 and CD103 and NK cells **(G)** modify receptors expression downmodulated CD47 and increasing CD90 marker, leading to IFNγ release.

Various cytokines/chemokines are involved in NK cell recruitment, including monocyte chemoattractant protein-1 (MCP-1/CCL2), fractalkine (CX3CL1), IL-15, IL-12, IL-18, and IFN-α (68, 69). Moreover, IL-15, IL-12, and IL-18 drive NK cell pro-atherogenic features, by hyper-activating NK cells, either in a direct manner or *via* dendritic cells (DC) and monocyte/macrophages interaction (70–72) (**Figure 1A**). Atherosclerotic plaques are enriched in IL-12 producing macrophages, in response to oxLDL (73) (**Figure 1B**). IL-12 within the ATS plaque enhances NK cell cytolytic activity *via* IFNγ production, resulting in plaque destabilization, through either the induction of smooth muscle cells (SMCs) apoptosis and/or secretion of matrix metalloproteinases (MMPs) (74). In addition, oxLDL supports the interaction between NK cells and DCs, in a CD48-2B4 contact-dependent manner **(** **Figure 1C** **)**. Opsonized LDL favors the NK-DC crosstalk, *via* IL-12 and IFNγ, resulting in altered DC editing/activation and/or selection of highly inflammatory M1-like macrophages (74) **(** **Figure 1D** **).** Both macrophages and endothelial cells within the ATS plaques have been reported to express NKG2D ligands that correlate with detectable serum levels of soluble Major Histocompatibility Complex (MHC) class I chain-related proteins A (sMICA), which in turn activated killing abilities in NK cells (75) **(** **Figure 1E** **).**

In human ATS, most of the current knowledge on NK cells comes from observational studies on lesion samples conducted by immunohistochemistry. As far as we know, phenotypic and functional features of NKs in ATS patients are still not completely known. In this context, a recent study from Bonaccorsi et al. characterized NK cells in atherosclerotic plaques of asymptomatic patients. They found that carotid plaques are enriched in CD56^bright^perforin^low^ NK cells, which also express tissue-resident markers, such as CD103, CD69 and CD49a (76), **(** **Figure 1F** **)** with increased production of IFNγ (76). These data suggest that hyperactivation of NK cells in carotid plaques may represent a relevant host-dependent mechanism determining plaque instability **(** **Figures 1F, G** **)**.

In line with this hypothesis, using a murine model of adeno-associated virus (AAV)-induced hypercholesterolemia, Engelbertsen et al. (77) showed that loss of CD47 results in increased frequency of IFNγ producing CD90^+^ NK cells (77) **(** **Figure 1G** **)**. Moreover, depletion of NK cells, using anti-NK1.1 monoclonal antibody, generates equalization of atherosclerotic burden, supporting that NK cell are involved in ATS progression in CD47-null mice (77). CD47 (an integrin-associated protein, IAP), is a transmembrane protein exerting multiple biological activities, ranging from regulation of efferocytosis to leukocyte trafficking (77, 78). There are contrasting reports, showing both pro-atherogenic and anti-atherogenic effects of CD47 and its ligands (77–79). Engelbertsen and colleagues (77) also found that CD90^+^ NKs are expanded in the atherosclerotic aorta and largely produce IFNγ, as compared to CD90^-^ NK cells (77).

Upregulation of cell cholesterol is a relevant hallmark of the ATS plaque, exacerbating the pro-inflammatory state that impact on the plaque fate, by continuously damaging the ECs and SMCs, finally determining the plaque rupture. Of notice, upregulated cell cholesterol induce a signature of trained immunity (80). Trained immunity, a concept recently developed, refers to the ability of innate immunity to develop a specific memory as for adaptive immunity (17). This innate immunological memory, leads to an augmented long-lasting proinflammatory immune response to a secondary stimulus, through metabolic and epigenetic rewiring of innate immune cells (17). Moreover, it has been demonstrated that, in addition to microbial stimuli, endogenous metabolites, such as oxLDL and lipoproteins, that characterize the plaque microenvironment, train the generation of pro-atherogenic monocytes and macrophages, by instructing these cells to produce ATS-supporting cytokines, such as GM-CSF, IFNγ, IL-3 and MMPs. Mechanistically, this immune training is sustained by a metabolical switch from oxidative phosphorylation (OxPhos) to aerobic glycolysis and mevalonate synthesis, in monocytes, together with epigenetic modifications (17, 81).

In the case of ATS and all those phatologies where persistent chronic inflammation has a detrimental role, trained immunity can be considered a double edge sword. ECs, apart from their vascular function, are now considered as relevant immunoregulatory effectors in chronic inflammatory diseases (82, 83). Following cellular damage, ECs release large amount of IL-1β and IL-6 and increase the expression of adhesion molecules, like E-selectin (CD62E) and ICAM-1 (CD54), that further contribute to the exacerbate inflammation and trained immunity (80), including in ATS. In the case of ATS, the persistent activation of the inflammatory state, due to trained immunity, has been hypothesized as a relevant mechanisms linking non-resolving inflammation in ATS (84–86). As for macrophages, given the shared immune cell plasticity and capabilities to adapt to different pathophysiological environments, NK cells could be considered as key innate immune cells within the ATS plaque, both as a “soloists” or by interacting with atherogenic macrophages. In this context, the microenvironment of cholesterol-mediated exposure may also trigger trained immunity on NK cells, exacerbating their activity in the plaque, mimicking the same effect observed in macrophages.

Therefore, studies investigating the contribution of NK cells to ATS and their specific polarization state in ATS, still require more investigation.

ATS plaques are enriched in cholesterol that, through enzymatic reactions or by auto-oxidation with reactive oxygen species (ROS), is converted into oxysterol (87). Oxysterols can modulate NK cell metabolism and subsequently activity by inhibition of Sterol regulatory element binding protein (SREBP), which is required for proper cytokine-induced growth and effector function by NK cells (88). Indeed, both glycolysis and oxidative phosphorylation (OxPhos) are SREBP-dependent mechanisms and NK cells that cannot activate SREBP showed reduced glucose metabolism and impaired effector functions (88). Within ATS plaques, the higher amount of oxysterols can reduce NK cell functionality by blocking SREBP activity.

Related to the presence of cholesterol within ATS plaque, the NOD-like Receptor Protein 3 (NLRP3), an inflammasome component (89), is another player involved in both ATS onset and development and NK cell modulation. NLRP3 inflammasome (89) is a multimeric protein complex that, upon caspase-1 activation, leads to the release of inflammatory cytokines IL-1β and IL-18 (90).

Duewell and colleagues (90) have shown how cholesterol crystals are able to activate NLRP3 inflammasome, already in early stages of ATS, inducing inflammation and how this condition is impaired in mice deficient in components of the NLRP3 inflammasome, also following cholesterol crystal intraperitoneal injection (90). Moreover, it has been shown that *LDLr*-/- mice, transplanted with bone marrow (BM) from NLRP3-deficient mice, display an impaired development of early atherosclerosis (89), confirming the involvement of NLRP3 in ATS onset.

Interesting, IL-18 release, upon NLRP3 inflammasome activation, can impact not only NK cell recruitment (as mentioned before), but it has been shown that can affect NK cell cytotoxicity in murine model of cancer (91). Indeed, mice deficient in NLRP3 inflammasome components show increased growth of liver colorectal cancer (CRC) metastasis, in a mechanism dependent on the lower level of IL-18 and by the subsequent reduction of hepatic NK cell cytotoxicity (91). This data suggested that NLRP3 activation in ATS plaque could mediate NK cells recruitment and activation by IL-18 release.

Opposite evidence derives from a study on hepatocellular carcinoma (HCC) where NLRP3 has been reported to be involved in cancer development. In this study, HCC patients showed reduction and impairment in NK cells. Using *in vitro* co-culture system of NK cell line NK-92 and HCC cells, it has been shown that NLRP3 down-modulation in HCC cells induces lowered expression of metalloproteinase, subsequent to MICA upregulation which in turn increases NK-92 toxicity, through NKG2D binding (92). This result is further confirmed by exploiting a xenograft mouse model in which NLRP3 knock-out (KO) in HCC cell delays cancer development, reduces metastasis formation, and increases NK cell toxicity, through MICA-NKG2D interaction (92).

MICA is a surface protein that, upon proteinases cleavage, becomes soluble (sMICA) (93). MICA is overexpressed in both macrophages and endothelial cells within the ATS plaque and showed same function as sMICA in promoting NK cell cytotoxicity (94). Considering reported data, we can speculate that NLRP3 activation, by cholesterol crystals, affects the shedding of MICA from macrophages and endothelial cells by proteinases upregulation, and that sMICA increases NK cells recruitment within ATS plaque.

Given the complexity of ATS plaque environment, the molecular and cellular players that modulate its onset and development, and the continuous remodeling of the ATS plaque, it is not surprising that contrasting data suggest a dichotomous role of NK cells in ATS onset and development.

### Risk Factors for ATS: NK Cells in Type-2 Diabetes

Diabetes mellitus represents one of the major risk factors for CVDs, including ATS (95). Both type 1 and type 2 diabetes are accompanied by micro and macrovascular complications. Type 2 diabetes (T2D), a chronic complex disorder characterized by de-regulated inflammation and metabolic alterations, represents the most common form of the disease and affects about 95% of the diabetic population (96, 97). A major feature of T2D is represented by a peripheral resistance to the action of insulin and a failure of beta cells to compensate for this alteration, resulting in hyperglycemia. The biology of both cardiovascular and immune cells is altered by chronic or transient hyperglycemia and the consequent increased oxidative stress with ROS accumulation/production.

In diabetes, ROS production is driven by mitochondrial respiration, in response to glucose stimulation (98). When peripheral insulin resistance impaired glucose clearance, the continuous glycolytic flux increases ROS production (99) that exerts different effects, according to NK cells phenotype in the inflammatory microenvironment. In diabetes, ROS production is mainly induced by hypoxia (98) that, as ROS, can act as NK cell modulator.

Indeed, hypoxia modulates NK cell metabolism promoting glycolysis and reducing OxPhos that leads to enhanced cytotoxicity and increased IFNγ production (99, 100). This hypoxia-related effect is also supported by MICA. Indeed, in inflammatory environment, such as in renal epithelial cells (101) and in cardiomyocytes (102), Hypoxia inducible factor-1α (HIF-1α) induces MICA upregulation that stimulates NK cytotoxicity and IFNγ production (94). The chronic excess of glucose induces visceral adipose tissue (VAT) expansion and dysfunctions mediated by adipocytes and immune cells that contribute to hypoxia (103). Thus, in T2D VAT, we can speculate that immune cells, including NK cells, are involved in disease progression by modulating VAT microenvironment that in turn support inflammation and immune cell recruitment, by different mechanisms, including hypoxia.

Of note, even though high glucose concentration predisposes to ATS development, the use of lowering glucose drugs does not result in the reduction of the CV risk in diabetic subjects, a concept known as metabolic memory (104). In the case of immune cells, the persistent changes due to environmental alteration in nutrition and metabolism are referred to as “training”. Emerging data are supporting the idea that trained immunity can be associated with diabetes and its damaging effect on the CV system (105). Concerning innate immunity, several reports demonstrated the role of monocyte/macrophages in the process of ATS and increasing myelopoiesis in the diabetic bone marrow, but less is known on NK cells (106–108). Of note, in subjects with diabetes and diabetic atherosclerotic complications, the BM is dramatically remodeled, including deposition of pro-inflammatory adipocytes, decreased innervation, and vascularization with associated impaired hematopoiesis (109–113). The number of BM resident NK cells is increased in diabetic patients with or without ischemic complications, but no changes in the circulating cell pool were observed (109, 114).

**Figure 2:**
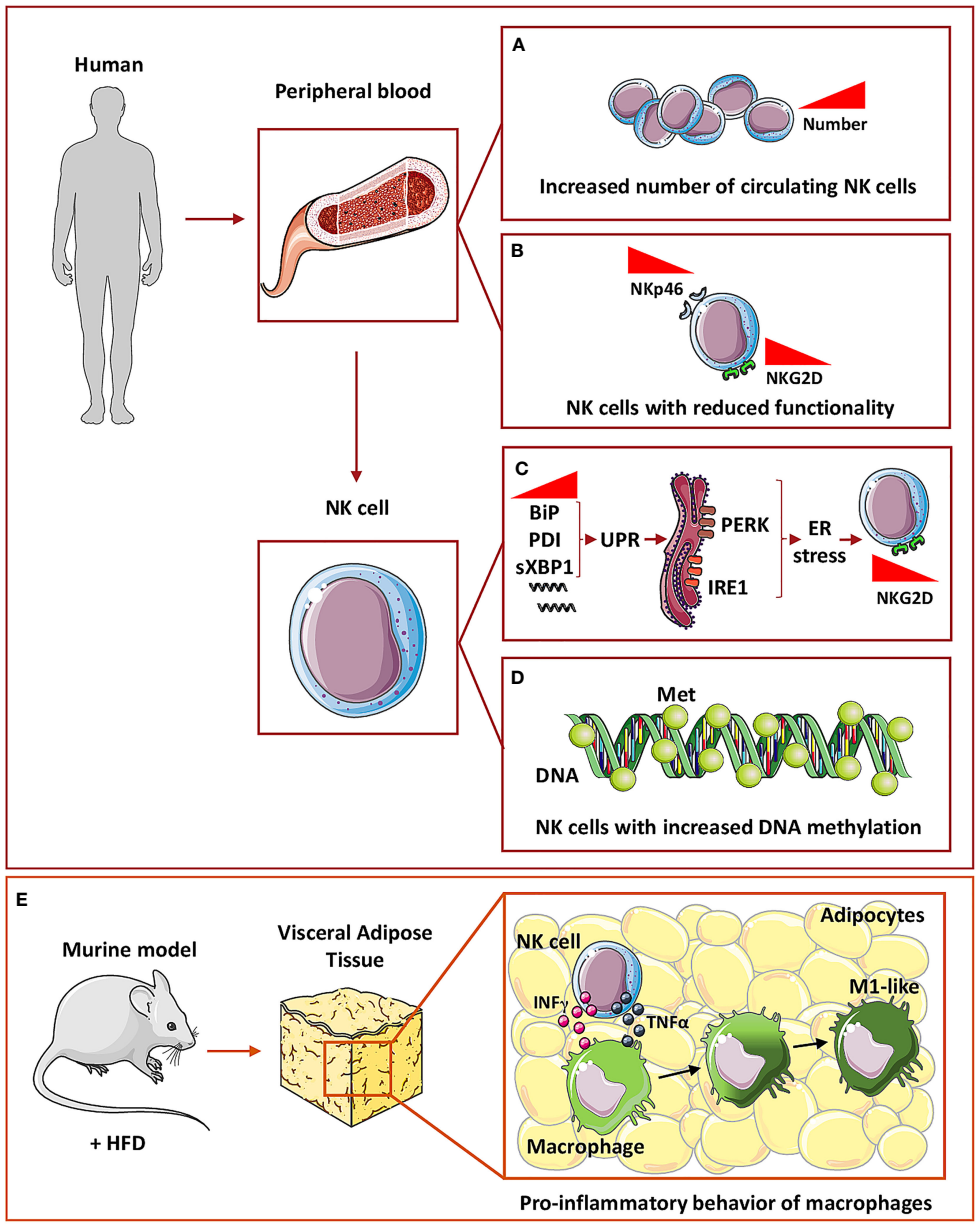
NK cells in Type 2 diabetes. In humans, circulating NK cells in T2D subjects have been found to increase in number **(A)** but with decreased expression of both NKp46 and NKG2D activation markers, thus showing a reduced functionality **(B)**. At the molecular level, circulating NK cells in T2D showed an increased mRNA expression of BiP, PDI, and sXBP1, a marker of unfolded protein response (UPR) which in turn is related to ER stress-activated by PERK and IRE1 sensors **(C)**. These mechanisms are related to NKG2D down-modulation in circulating NK cells **(C)**. In addition, NK cells showed an increased general DNA methylation **(D)**. To mimic T2D mice are fed with high fat diet (HFD) **(E)** and within visceral adipose tissue, NK cells through IFNγ and TNFα release can induce macrophages polarization toward a pro-inflammatory M1-like phenotype thus promoting inflammation **(E)**.

A recently published meta-analysis, collecting results from 13 independent studies, showed that circulating NK cells number increase in T2D patients (n=491), compared to healthy subjects (n=1607) (115) **(** **Figure 2A** **)**. However, other studies showed no differences in the phenotype between NK cells in T2D and NK cells in healthy controls (116). Hyperglycemia, sedentary lifestyle, poor metabolic status, all represent peculiar features of diabetes and have been reported to reduce NK cell functionality (117). Functional alteration of NK cells, isolated from T2D patients, include reduced expression of NKp46 and NKG2D receptors, decreased cytotoxic activity *in vitro*, compared to healthy subjects (118) **(** **Figure 2B** **)**. However, exposure to IL-15, a major cytokine involved in NK cell re-education and activation, restored NK cell functionality in T2D patients (117). Mechanistically, it has been demonstrated that endoplasmic reticulum (ER) stress, which is a crucial mediator of diabetes-associated complications, is induced by tunicamycin, a mediator for the unfolded protein response (UPR), with subsequent reduction of NKG2D and NKp46 expression (117). In addition, markers of UPR, such as BiP, PDI, and sXBP1, are increased in NK cells from T2D patients and ER stress is activated through PERK and IRE1 sensors, which are involved in UPR and that are causative of NKG2D down-modulation in NK cells from T2D patients (117) **(** **Figure 2C** **)**.

Also, NKG2D expression in NK cells was found to be negatively correlated with glycated hemoglobin (HbA1c) level, suggesting that hyperglycemia could directly govern NK cell functional alterations (117). A correlation between hyperglycemia and NK cells activity has been also demonstrated by Kim et al. (119). In a recent study, Kim and colleagues (119) enrolled 49 participants, 21 with T2D, 15 with pre-diabetes, and 13 controls with normal glucose tolerance, to analyze NK cells activity modulation, as related to the diabetes stage. NK cells activity was measured by detecting circulating IFNγ level together with HbA1c. They showed that HbA1c displayed an inverse linear correlation with NK cells activity (119), together with diabetes progression and they conclude that HbA1c is an independent predictor of NK cell activity in T2D patients (119).

Moreover, epigenetic alterations functionally impact on immune cell effectors (120). While no global DNA methylation was observed in peripheral blood mononuclear cells, monocytes, lymphocytes, or T cells, NK cells from T2D patients exhibit increased methylation levels that positively correlate with insulin resistance, linking DNA methylation changes, immune cell function, and metabolic dysfunction (120) **(** **Figure 2D** **)**.

Finally, it has been reported that within VAT of mice receiving HFD, NK cells support the development of obesity-induced insulin resistance, *via* induction of pro-inflammatory/M1-like macrophages, through a mechanism mediated by NK-derived cytokines, including TNFα (121) **(** **Figure 2E** **)**.

### Risk Factors for ATS: NK Cells in Obesity

Obesity drives a program of a chronic pro-inflammatory state that orchestrates the development of related co-morbidities, including cancer, T2D, CVDs, and ATS (122–125). Several pro-inflammatory cytokines are aberrantly produced in obese individuals and de-regulate the normal homeostasis, such as IL-1 (interacting with insulin signaling) and IL-17 (interacting with adipogenesis) (126, 127). Obesity is widely recognized as a pivotal risk factor for T2D, as a consequence of the induction of chronic low-grade inflammation in local adipose tissue (128).

Immuno-metabolic alterations characterizing obese individuals significantly impact on NK cell functions. Resting NK cells metabolize glucose, *via* glycolysis, coupled to oxidative phosphorylation, yielding high levels of energy (99, 129–132). Following activation, NK cells rapidly increase their rates of aerobic glycolytic metabolism, providing the biosynthetic precursors for cytokine and lytic granule production (129–131).

**Figure 3:**
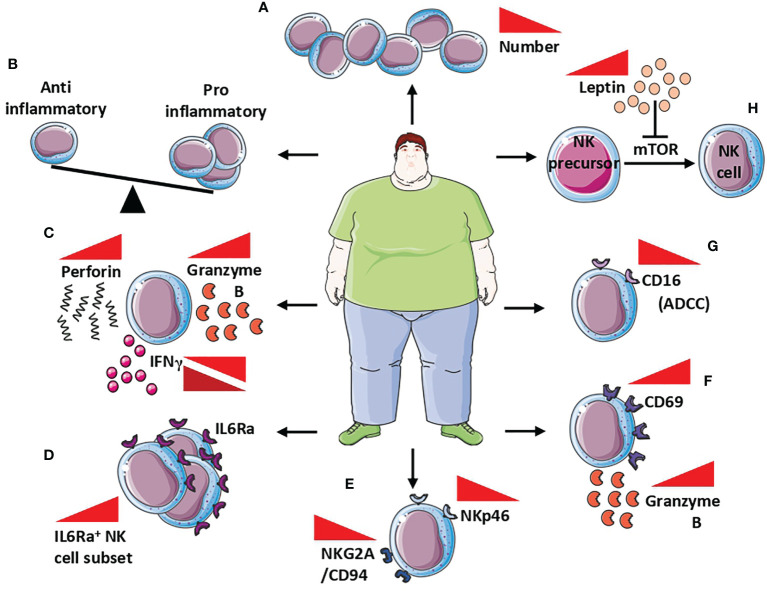
NK cells in obesity. Alteration of NK cells in obese patients includes **(A)** reduced NK cell frequency, **(B)** unbalance between anti- and pro-inflammatory behavior of NK cells, and **(C)** increased release of both perforin and granzymes B, while IFNγ is found both up and down modulated. At the molecular level, NK cells of obese patients showed a **(D)** increased number of IL6Rα+ cells that correlated with low-grade inflammation markers, **(E)** decreased expression of NKp46 and NKG2A/CD94, with subsequently reduced cytotoxicity, **(F)** increased expression CD69 activation marker correlated with increased release of granzyme B, and **(G)** reduction of CD16 responsible for the reduced ADCC. Finally, the increased circulating level of leptin in obese patients **(H)** affects NK cells maturation by the inhibition of mTOR signalling in NK cells precursors, resulting in reduced maturation, thus contributing to reduced NK cell frequency.

Obese patients showed decreased NK cell frequency **(** **Figure 3A** **)**; in these patients, NK are characterized by pro-inflammatory functions **(** **Figure 3B** **)**, and unbalance in the equilibrium between inhibitory and activation receptors, lytic capabilities, the release of perforin/granzymes, and altered release of IFNγ (130) **(** **Figure 3C** **)**. Interestingly, alterations in NK cell frequency (reduced circulating NK cells number) have been found also in obese children, in an insulin resistance-dependent manner (133).

Circulating levels of IL-6 are strongly elevated in obese individuals (134). IL-6 receptor (IL-6R) expression was found in blood, hepatic, adipose tissue, and splenic NK cells, in mice as well as in the human NK-92 cell line and NK cells isolated from blood, spleen, and tonsil of humans (135–139). In a murine model, Theurich et al. (137) demonstrated that obesity promotes the expansion of a distinct IL6R^+^CSF1R^+^ NK cell subset, that further supports obesity **(** **Figure 3D** **)**. Selective ablation of this NK cell subpopulation prevents obesity and insulin resistance. Moreover, conditional inactivation of IL-6Rα or STAT3 in NK cells limits obesity-associated induction by NK cells, protecting from obesity, insulin resistance, and obesity-associated inflammation (137). Results from Theurich et al. (137) were also confirmed in humans, where IL6Rα^+^ NK cells increase in obese patients and correlates with markers of systemic low-grade inflammation (137).

Peripheral blood NKs in obese patients are endowed with phenotype and functional alterations, characterized by reduced expression of the natural cytotoxicity receptor NKp46 and the NKG2A/CD94 complex, which was correlated with body mass index (BMI) (140) **(** **Figure 3E** **)**. While expressing high levels of the activation marker CD69 and granzyme B **(** **Figure 3F** **)**, peripheral blood NKs from obese patients have very low expression of the CD16 antigen, which is responsible for the reduced ADCC (131) **(** **Figure 3G** **)**. BMI inversely correlates with the impaired capability of peripheral blood NK cells to degranulate or to produce MIP-1β, following co-culture with K562 cells (131).

Leptin and adiponectin production is altered during obesity and these adipokines have been largely reported to modulate NK cell activities (141). Leptin is able to inhibit mTOR, whose activation is necessary for NK cell maturation, by regulating their responsivity to IL-2 and IL-15 (131, 142, 143) **(** **Figure 3H** **)**. Since IL-15 is produced by adipocytes (144, 145), it should be hypothesized that leptin impairment, occurring during obesity, is directly involved in NK cell hyporesponsiveness in an mTOR-dependent manner.

Also, adipocytes have been reported to regulate immune response in cancer patients with obesity (123, 146, 147). The connection between adipocyte-derived leptin and immunomodulation in NK cells, has been demonstrated *in vitro* and *in vivo*.

*In vitro*, cytolytic NK cells, exposed to leptin, exhibit decreased ability to release IFNγ and were less effective in lysing colorectal cancer (CRC) cell lines (129).

In an *in vivo* model of chemically-induced CRC carcinogenesis by azoxymethane (AOM), mice receiving a HFD showed decreased frequency and functionality of NK cells in the spleen and the livers (129).

In addition, in VAT of obese patients, NLRP3 inflammasome is upregulated and *in vivo* experiments showed that NLRP3 deficient mice are protected from HFD-induced obesity (89), suggesting a crucial role of inflammation in obesity. The link between inflammation and obesity is further confirmed by other evidence as free fatty acids, increased in obesity, can modulate inflammation.

Palmitate, in HFD fed mice, can activate NLRP3 inflammasome in adipose tissue macrophages, increasing IL-1β and IL-18 secretion (148). Also, adipose tissue macrophages are polarized toward M1 phenotype and secrete inflammatory cytokines, such as IL-1β, IL-6, and TNF-α that, in turn, can recruit and stimulate NK cells (148).

Obesity arises also from compromised adipose storage that drives inflammation within VAT. This evidence, together with the reported data, support the idea that a vicious loop between obesity and inflammation is crucial for disease onset and progression and, within this loop, NK cells could play a role as a contributor to pro-inflammatory environment development.

These results further corroborate the direct link between obesity, a risk factor for ATS, with NK cell activities.

## Conclusions and Future Perspectives

The immune system, whose native functions are protecting the host, acts as a double-edged sword in the control and insurgence/progression of chronic pathologic disorders, as a consequence of immune cell plasticity. Inflammation is a common hallmark of CVDs, including ATS and ATS-associated risk factors, such as obesity, and T2D. These pathological conditions are characterized by a chronic low-grade inflammation that is involved in regulating immune cell plasticity and adaptation capabilities to the pathophysiological environment(s) of the host organism. Thus modifying/re-educating the immune system, represents a challenging new therapeutic approach. Emerging cancer immunotherapy with engineered T-cells is a successful example of this clinical translation. Of note, NK cells represent a perfect tool for immunotherapy, due to their natural capability to reach/recognize cells that “have to be eliminated”. Immunotherapies, based on targeting and/or using NK cells, have been explored and include re-education with cocktails of activating (immune)-cytokines (IL-2, IL-12, IL-15), adoptive transfer of modified (BiKe, TriKe) NKs and generation of CAR-NKs. Considering that inflammation and immune cell plasticity are crucial hallmarks of both cancers and CVDs, the relevant question now is whether immunotherapy can be translated to CVDs. Recently, the contribution of diverse subpopulations of immune cells, beyond monocytes and macrophages, to CVDs, has been intensively investigated. Moreover, recent clinical trials have employed biological modifiers of proinflammatory cytokine function, such as targeting TNFα and IL-1β, in heart failure. Surprisingly, little is known about the role of NK cells. We here summarized the current knowledge on NK cells contribution to ATS, and to ATS-associated risk factors, such as T2D, stressing the need of acquiring more information in these fields, to enable designing future NK cells-oriented therapeutic approaches, including re-educating them, according to the CVD pathological setting and environments.

## Author Contributions

Conceptualization: MTP, GS, and AB. Text drafting and editing: MTP, MC, MG, FR, LM, GG, GA, GS, and AB. Critical revision: MTP, LM, GG, GA, GS, and AB. Figure preparation: MTP and MC. Funds: AB. All authors contributed to the article and approved the submitted version.

## Funding

This work has been supported by the Italian Ministry of Health Ricerca Corrente-IRCCS MultiMedica, for GS, GG and AB. AB is recipient of a research grant funded by the Italian Association for Cancer Research (AIRC-MFAG, ID 22818) and a research grant funded by the Cariplo Foundation (ID 2019-1609). MG is a participant to PhD course in Life Sciences and Biotechnology at the University of Insubria, Varese, Italy and funded by the Italian Ministry of University and Research PRIN 2017 (ID: 2017NTK4HY). MC is a participant to PhD course in Experimental and Translational Medicine at the University of Insubria, Varese, Italy.

## Conflict of Interest

The authors declare that the research was conducted in the absence of any commercial or financial relationships that could be construed as a potential conflict of interest.

## Publisher’s Note

All claims expressed in this article are solely those of the authors and do not necessarily represent those of their affiliated organizations, or those of the publisher, the editors and the reviewers. Any product that may be evaluated in this article, or claim that may be made by its manufacturer, is not guaranteed or endorsed by the publisher.

## Abbreviations

AAV, adeno associated virus; ADCC, antibody dependent cellular cytotoxicity; AOM, azoxymethane; ATS, atherosclerosis; BiP, binding immunoglobulin protein; BMI, body mass index; CAR, chimeric antigen receptor; CRC, colorectal cancer; CSF1R, colony-stimulating factor 1 receptor; CXCL, chemokine ligand (C-X-C motif); CVDs, cardiovascular diseases; DC, dendritic cell; dNK cells, decidual Natural Killer cells; ECs, endothelial cells; EOMES, eomesodermin; ER, endoplasmic reticulum; FOXP3, forkhead box P3; GM-1, ganglioside monosialic type 1; HbA1c, gycosylated hemoglobin; HFD, high fat diet; HIF-1, hypoxia inducible factor 1; IAP, integrin associated protein; IFNγ, interferon γ; IL-, interleukin-; ILCs, innate lymphoid cells; IRE1, inositol-requiring enzyme 1; LADA, latent autoimmune diabetes in adults; MCP-1/CCL2, monocyte chemoattractant protein-1; MHC, major histocompatibility complex; MICA, MHC class I chain-related protein A; MIP1-β, macrophage inflammatory protein 1-β; MPs, matrix metalloproteinases; mTOR, mammalian target of rapamycin; NCAM, neural cell adhesion molecule; NCR, natural cytotoxicity receptor; NK, natural killer; NKG2A/D, Natural Killer receptor Group 2 A/D; NOD, non-obese diabetic; NLRP3, NOD-like Receptor Protein 3; oxLDL, Oxidized Low-Density Lipoprotein; OxPhos, Oxidative Phosphorylation; PB, Peripheral Blood; PDI, Protein Disulfide-Isomerase; PERK, Protein kinase R (PKR)-like Endoplasmic Reticulum Kinase; PlGF, Placental Growth Factor; RAE1, Retinoic Acid Early transcript 1; RAG, Recombinant Activating Gene; RIP, Rat Insulin Promoter; ROS, Reactive Oxygen Species; SREBP, Sterol regulatory Element Binding Protein; SMCs, Smooth Muscle Cells; sMICA, soluble MHC class I Chain-related Protein A; STAT, Signal Transducer and Activator of Transcription; sXBP1, spliced X-Box Binding Protein 1; TANKs, Tumor Associated Natural Killer cells; T-bet, T-box expressed in T cells; TCR, T Cell Receptor; TG, Transgenic; TINKs, Tumor Infiltrating NK cells; TNFα, TumorNecrosis Factor-α; T2D, Type 2 Diabetes; UPR, Unfolded Protein Response; VEGF, Vascular Endothelial Growth Factor.

## References

[1] Ruparelia N Chai JT Fisher EA Choudhury RP. Inflammatory Processes in Cardiovascular Disease: A Route to Targeted Therapies. Nat Rev Cardiol (2017) 14(3):133–44. doi: 10.1038/nrcardio.2016.185 PMC5525550 27905474

[2] Moore KJ. Targeting Inflammation in CVD: Advances and Challenges. Nat Rev Cardiol (2019) 16(2):74–5. doi: 10.1038/s41569-018-0144-3 PMC6392425 30560921

[3] Kaplan MJ. Management of Cardiovascular Disease Risk in Chronic Inflammatory Disorders. Nat Rev Rheumatol (2009) 5(4):208–17. doi: 10.1038/nrrheum.2009.29 19337285

[4] Swirski FK Nahrendorf M. Cardioimmunology: The Immune System in Cardiac Homeostasis and Disease. Nat Rev Immunol (2018) 18(12):733–44. doi: 10.1038/s41577-018-0065-8 30228378

[5] Lazzerini PE Hamilton RM Boutjdir M. Editorial: Cardioimmunology: Inflammation and Immunity in Cardiovascular Disease. Front Cardiovasc Med (2019) 6:181. doi: 10.3389/fcvm.2019.00181 31850376 PMC6901670

[6] Baci D Bosi A Parisi L Buono G Mortara L Ambrosio G. Innate Immunity Effector Cells as Inflammatory Drivers of Cardiac Fibrosis. Int J Mol Sci (2020) 21(19). doi: 10.3390/ijms21197165 PMC7583949 32998408

[7] Wang J Duan Y Sluijter JP Xiao J. Lymphocytic Subsets Play Distinct Roles in Heart Diseases. Theranostics (2019) 9(14):4030–46. doi: 10.7150/thno.33112 PMC6592175 31281530

[8] Frostegard J. Immunity, Atherosclerosis and Cardiovascular Disease. BMC Med (2013) 11:117. doi: 10.1186/1741-7015-11-117 23635324 PMC3658954

[9] Wolf D Ley K. Immunity and Inflammation in Atherosclerosis. Circ Res (2019) 124(2):315–27. doi: 10.1161/CIRCRESAHA.118.313591 PMC6342482 30653442

[10] Ridker PM Everett BM Thuren T MacFadyen JG Chang WH Ballantyne C. Antiinflammatory Therapy With Canakinumab for Atherosclerotic Disease. N Engl J Med (2017) 377(12):1119–31. doi: 10.1056/NEJMoa1707914 28845751

[11] Nidorf SM Eikelboom JW Budgeon CA Thompson PL. Low-Dose Colchicine for Secondary Prevention of Cardiovascular Disease. J Am Coll Cardiol (2013) 61(4):404–10. doi: 10.1016/j.jacc.2012.10.027 23265346

[12] Meijers WC Maglione M Bakker SJL Oberhuber R Kieneker LM de Jong S. Heart Failure Stimulates Tumor Growth by Circulating Factors. Circulation (2018) 138(7):678–91. doi: 10.1161/CIRCULATIONAHA.117.030816 29459363

[13] Koene RJ Prizment AE Blaes A Konety SH. Shared Risk Factors in Cardiovascular Disease and Cancer. Circulation (2016) 133(11):1104–14. doi: 10.1161/CIRCULATIONAHA.115.020406 PMC4800750 26976915

[14] Galli SJ Borregaard N Wynn TA. Phenotypic and Functional Plasticity of Cells of Innate Immunity: Macrophages, Mast Cells and Neutrophils. Nat Immunol (2011) 12(11):1035–44. doi: 10.1038/ni.2109 PMC3412172 22012443

[15] Almeida FF Belz GT. Innate Lymphoid Cells: Models of Plasticity for Immune Homeostasis and Rapid Responsiveness in Protection. Mucosal Immunol (2016) 9(5):1103–12. doi: 10.1038/mi.2016.64 27484190

[16] Hirahara K Poholek A Vahedi G Laurence A Kanno Y Milner JD. Mechanisms Underlying Helper T-Cell Plasticity: Implications for Immune-Mediated Disease. J Allergy Clin Immunol (2013) 131(5):1276–87. doi: 10.1016/j.jaci.2013.03.015 PMC3677748 23622118

[17] Netea MG Dominguez-Andres J Barreiro LB Chavakis T Divangahi M Fuchs E. Defining Trained Immunity and Its Role in Health and Disease. Nat Rev Immunol (2020) 20(6):375–88. doi: 10.1038/s41577-020-0285-6 PMC7186935 32132681

[18] Parisi L Bassani B Tremolati M Gini E Farronato G Bruno A. Natural Killer Cells in the Orchestration of Chronic Inflammatory Diseases. J Immunol Res (2017) 2017:4218254. doi: 10.1155/2017/4218254 28428965 PMC5385901

[19] Parisi L Gini E Baci D Tremolati M Fanuli M Bassani B. Macrophage Polarization in Chronic Inflammatory Diseases: Killers or Builders? J Immunol Res (2018) 2018:8917804. doi: 10.1155/2018/8917804 29507865 PMC5821995

[20] Gieseck RL 3rd Wilson MS Wynn TA. Type 2 Immunity in Tissue Repair and Fibrosis. Nat Rev Immunol (2018) 18(1):62–76. doi: 10.1038/nri.2017.90 28853443

[21] Boulter L Bullock E Mabruk Z Brunton VG. The Fibrotic and Immune Microenvironments as Targetable Drivers of Metastasis. Br J Cancer (2021) 124(1):27–36. doi: 10.1038/s41416-020-01172-1 33239677 PMC7782519

[22] Zouali M La Cava A. Editorial: Innate Immunity Pathways in Autoimmune Diseases. Front Immunol (2019) 10:1245. doi: 10.3389/fimmu.2019.01245 31214194 PMC6557999

[23] Shi FD Ljunggren HG Sarvetnick N. Innate Immunity and Autoimmunity: From Self-Protection to Self-Destruction. Trends Immunol (2001) 22(2):97–101. doi: 10.1016/s1471-4906(00)01821-4 11286711

[24] Sasaki K Himeno A Nakagawa T Sasaki Y Kiyonari H Iwai K. Modulation of Autoimmune Pathogenesis by T Cell-Triggered Inflammatory Cell Death. Nat Commun (2019) 10(1):3878. doi: 10.1038/s41467-019-11858-7 31462647 PMC6713751

[25] Gummlich L. Tumours Disrupt the Immune Scenery. Nat Rev Cancer (2020) 20(8):415. doi: 10.1038/s41568-020-0286-6 32620876

[26] Botta C Misso G Martino EC Pirtoli L Cusi MG Tassone P. The Route to Solve the Interplay Between Inflammation, Angiogenesis and Anti-Cancer Immune Response. Cell Death Dis (2016) 7:e2299. doi: 10.1038/cddis.2016.211 27441651 PMC4973351

[27] Bruno A Pagani A Pulze L Albini A Dallaglio K Noonan DM. Orchestration of Angiogenesis by Immune Cells. Front Oncol (2014) 4:131. doi: 10.3389/fonc.2014.00131 25072019 PMC4078768

[28] Libby P Ridker PM Maseri A. Inflammation and Atherosclerosis. Circulation (2002) 105(9):1135–43. doi: 10.1161/hc0902.104353 11877368

[29] Wu MY Li CJ Hou MF Chu PY. New Insights Into the Role of Inflammation in the Pathogenesis of Atherosclerosis. Int J Mol Sci (2017) 18(10). doi: 10.3390/ijms18102034 PMC5666716 28937652

[30] Olson NC Doyle MF Sitlani CM de Boer IH Rich SS Huber SA. Associations of Innate and Adaptive Immune Cell Subsets With Incident Type 2 Diabetes Risk: The MESA Study. J Clin Endocrinol Metab (2020) 105(3). doi: 10.1210/clinem/dgaa036 PMC7049263 31990975

[31] Olson NC Sitlani CM Doyle MF Huber SA Landay AL Tracy RP. Innate and Adaptive Immune Cell Subsets as Risk Factors for Coronary Heart Disease in Two Population-Based Cohorts. Atherosclerosis (2020) 300:47–53. doi: 10.1016/j.atherosclerosis.2020.03.011 32209232 PMC7276206

[32] Selathurai A Deswaerte V Kanellakis P Tipping P Toh BH Bobik A. Natural Killer (NK) Cells Augment Atherosclerosis by Cytotoxic-Dependent Mechanisms. Cardiovasc Res (2014) 102(1):128–37. doi: 10.1093/cvr/cvu016 24469537

[33] Whitman SC Rateri DL Szilvassy SJ Yokoyama W Daugherty A. Depletion of Natural Killer Cell Function Decreases Atherosclerosis in Low-Density Lipoprotein Receptor Null Mice. Arterioscler Thromb Vasc Biol (2004) 24(6):1049–54. doi: 10.1161/01.ATV.0000124923.95545.2c 14988092

[34] Abel AM Yang C Thakar MS Malarkannan S. Natural Killer Cells: Development, Maturation, and Clinical Utilization. Front Immunol (2018) 9:1869. doi: 10.3389/fimmu.2018.01869 30150991 PMC6099181

[35] Shi FD Ljunggren HG La Cava A Van Kaer L. Organ-Specific Features of Natural Killer Cells. Nat Rev Immunol (2011) 11(10):658–71. doi: 10.1038/nri3065 PMC3620656 21941294

[36] Huntington ND Vosshenrich CA Di Santo JP. Developmental Pathways That Generate Natural-Killer-Cell Diversity in Mice and Humans. Nat Rev Immunol (2007) 7(9):703–14. doi: 10.1038/nri2154 17717540

[37] Angus KL Griffiths GM. Cell Polarisation and the Immunological Synapse. Curr Opin Cell Biol (2013) 25(1):85–91. doi: 10.1016/j.ceb.2012.08.013 22990072 PMC3712171

[38] Smith SL Kennedy PR Stacey KB Worboys JD Yarwood A Seo S. Diversity of Peripheral Blood Human NK Cells Identified by Single-Cell RNA Sequencing. Blood Adv (2020) 4(7):1388–406. doi: 10.1182/bloodadvances.2019000699 PMC7160259 32271902

[39] Crinier A Milpied P Escaliere B Piperoglou C Galluso J Balsamo A. High-Dimensional Single-Cell Analysis Identifies Organ-Specific Signatures and Conserved NK Cell Subsets in Humans and Mice. Immunity (2018) 49(5):971–86.e5. doi: 10.1016/j.immuni.2018.09.009 30413361 PMC6269138

[40] Ni J Wang X Stojanovic A Zhang Q Wincher M Buhler L. Single-Cell RNA Sequencing of Tumor-Infiltrating NK Cells Reveals That Inhibition of Transcription Factor HIF-1alpha Unleashes NK Cell Activity. Immunity (2020) 52(6):1075–87.e8. doi: 10.1016/j.immuni.2020.05.001 32445619

[41] Yang C Siebert JR Burns R Gerbec ZJ Bonacci B Rymaszewski A. Heterogeneity of Human Bone Marrow and Blood Natural Killer Cells Defined by Single-Cell Transcriptome. Nat Commun (2019) 10(1):3931. doi: 10.1038/s41467-019-11947-7 31477722 PMC6718415

[42] Vitale M Caligiuri MA Sivori S. Editorial: Natural Killer Cells in Tissue Compartments. Front Immunol (2020) 11:258. doi: 10.3389/fimmu.2020.00258 32153578 PMC7044183

[43] Mikulak J Bruni E Oriolo F Di Vito C Mavilio D. Hepatic Natural Killer Cells: Organ-Specific Sentinels of Liver Immune Homeostasis and Physiopathology. Front Immunol (2019) 10:946. doi: 10.3389/fimmu.2019.00946 31114585 PMC6502999

[44] Peng H Tian Z. Diversity of Tissue-Resident NK Cells. Semin Immunol (2017) 31:3–10. doi: 10.1016/j.smim.2017.07.006 28802693

[45] Bassani B Baci D Gallazzi M Poggi A Bruno A Mortara L. Natural Killer Cells as Key Players of Tumor Progression and Angiogenesis: Old and Novel Tools to Divert Their Pro-Tumor Activities Into Potent Anti-Tumor Effects. Cancers (Basel) (2019) 11(4). doi: 10.3390/cancers11040461 PMC6521276 30939820

[46] Hanna J Goldman-Wohl D Hamani Y Avraham I Greenfield C Natanson-Yaron S. Decidual NK Cells Regulate Key Developmental Processes at the Human Fetal-Maternal Interface. Nat Med (2006) 12(9):1065–74. doi: 10.1038/nm1452 16892062

[47] Blois SM Klapp BF Barrientos G. Decidualization and Angiogenesis in Early Pregnancy: Unravelling the Functions of DC and NK Cells. J Reprod Immunol (2011) 88(2):86–92. doi: 10.1016/j.jri.2010.11.002 21227511

[48] Bosi A Zanellato S Bassani B Albini A Musco A Cattoni M. Natural Killer Cells From Malignant Pleural Effusion Are Endowed With a Decidual-Like Proangiogenic Polarization. J Immunol Res (2018) 2018:2438598. doi: 10.1155/2018/2438598 29713652 PMC5896269

[49] Bruno A Bassani B D'Urso DG Pitaku I Cassinotti E Pelosi G. Angiogenin and the MMP9-TIMP2 Axis Are Up-Regulated in Proangiogenic, Decidual NK-Like Cells From Patients With Colorectal Cancer. FASEB J (2018) 32(10):5365–77. doi: 10.1096/fj.201701103R 29763380

[50] Bruno A Ferlazzo G Albini A Noonan DM. A Think Tank of TINK/TANKs: Tumor-Infiltrating/Tumor-Associated Natural Killer Cells in Tumor Progression and Angiogenesis. J Natl Cancer Inst (2014) 106(8):dju200. doi: 10.1093/jnci/dju200 25178695 PMC4344546

[51] Bruno A Focaccetti C Pagani A Imperatori AS Spagnoletti M Rotolo N. The Proangiogenic Phenotype of Natural Killer Cells in Patients With non-Small Cell Lung Cancer. Neoplasia (2013) 15(2):133–42. doi: 10.1593/neo.121758 PMC3579316 23441128

[52] Bald T Krummel MF Smyth MJ Barry KC. The NK Cell-Cancer Cycle: Advances and New Challenges in NK Cell-Based Immunotherapies. Nat Immunol (2020) 21(8):835–47. doi: 10.1038/s41590-020-0728-z PMC8406687 32690952

[53] Cozar B Greppi M Carpentier S Narni-Mancinelli E Chiossone L Vivier E. Tumor-Infiltrating Natural Killer Cells. Cancer Discov (2020). doi: 10.1158/2159-8290.CD-20-0655 PMC7611243 33277307

[54] Kucuksezer UC Aktas Cetin E Esen F Tahrali I Akdeniz N Gelmez MY. The Role of Natural Killer Cells in Autoimmune Diseases. Front Immunol (2021) 12:622306. doi: 10.3389/fimmu.2021.622306 33717125 PMC7947192

[55] Libby P Theroux P. Pathophysiology of Coronary Artery Disease. Circulation (2005) 111(25):3481–8. doi: 10.1161/CIRCULATIONAHA.105.537878 15983262

[56] Bobryshev YV Lord RS. Identification of Natural Killer Cells in Human Atherosclerotic Plaque. Atherosclerosis (2005) 180(2):423–7. doi: 10.1016/j.atherosclerosis.2005.01.046 15910872

[57] Kosierkiewicz TA Factor SM Dickson DW. Immunocytochemical Studies of Atherosclerotic Lesions of Cerebral Berry Aneurysms. J Neuropathol Exp Neurol (1994) 53(4):399–406. doi: 10.1097/00005072-199407000-00012 8021714

[58] Cheng F Twardowski L Reifenberg K Winter K Canisius A Pross E. T and NK Cell Deficiency Accelerates Atherosclerosis in BALB/c Mice. PloS One (2016) 11(8):e0157311. doi: 10.1371/journal.pone.0157311 27564380 PMC5001715

[59] Winkels H Ley K. Natural Killer Cells at Ease: Atherosclerosis Is Not Affected by Genetic Depletion or Hyperactivation of Natural Killer Cells. Circ Res (2018) 122(1):6–7. doi: 10.1161/CIRCRESAHA.117.312289 29301835 PMC5761686

[60] Clerc G Rouz PM. Lymphocyte Subsets in Severe Atherosclerosis Before Revascularization. Ann Intern Med (1997) 126(12):1004–5. doi: 10.7326/0003-4819-126-12-199706150-00028 9182465

[61] Paigen B Ishida BY Verstuyft J Winters RB Albee D. Atherosclerosis Susceptibility Differences Among Progenitors of Recombinant Inbred Strains of Mice. Arteriosclerosis (1990) 10(2):316–23. doi: 10.1161/01.atv.10.2.316 2317166

[62] Schiller NK Boisvert WA Curtiss LK. Inflammation in Atherosclerosis: Lesion Formation in LDL Receptor-Deficient Mice With Perforin and Lyst(beige) Mutations. Arterioscler Thromb Vasc Biol (2002) 22(8):1341–6. doi: 10.1161/01.atv.0000024082.46387.38 12171798

[63] Nour-Eldine W Joffre J Zibara K Esposito B Giraud A Zeboudj L. Genetic Depletion or Hyperresponsiveness of Natural Killer Cells Do Not Affect Atherosclerosis Development. Circ Res (2018) 122(1):47–57. doi: 10.1161/CIRCRESAHA.117.311743 29046274

[64] Nishikado H Mukai K Kawano Y Minegishi Y Karasuyama H. NK Cell-Depleting Anti-Asialo GM1 Antibody Exhibits a Lethal Off-Target Effect on Basophils *In Vivo* . J Immunol (2011) 186(10):5766–71. doi: 10.4049/jimmunol.1100370 21490162

[65] Santoni A Peterson ES Knott DC Overton WR Herberman RB Holden HT. Reactivity of Anti-Asialo GM1 Serum With Tumoricidal and Non-Tumoricidal Mouse Macrophages. J Leukoc Biol (1985) 37(5):597–614. doi: 10.1002/jlb.37.5.597 3884721

[66] Trambley J Bingaman AW Lin A Elwood ET Waitze SY Ha J. Asialo GM1(+) CD8(+) T Cells Play a Critical Role in Costimulation Blockade-Resistant Allograft Rejection. J Clin Invest (1999) 104(12):1715–22. doi: 10.1172/JCI8082 PMC409885 10606625

[67] Lee U Santa K Habu S Nishimura T. Murine Asialo GM1+CD8+ T Cells as Novel Interleukin-12-Responsive Killer T Cell Precursors. Jpn J Cancer Res (1996) 87(5):429–32. doi: 10.1111/j.1349-7006.1996.tb00241.x PMC5921113 8641977

[68] Allavena P Bianchi G Zhou D van Damme J Jilek P Sozzani S. Induction of Natural Killer Cell Migration by Monocyte Chemotactic Protein-1, -2 and -3. Eur J Immunol (1994) 24(12):3233–6. doi: 10.1002/eji.1830241249 7805752

[69] Umehara H Bloom ET Okazaki T Nagano Y Yoshie O Imai T. Fractalkine in Vascular Biology: From Basic Research to Clinical Disease. Arterioscler Thromb Vasc Biol (2004) 24(1):34–40. doi: 10.1161/01.ATV.0000095360.62479.1F 12969992

[70] Chistiakov DA Sobenin IA Orekhov AN Bobryshev YV. Dendritic Cells in Atherosclerotic Inflammation: The Complexity of Functions and the Peculiarities of Pathophysiological Effects. Front Physiol (2014) 5:196. doi: 10.3389/fphys.2014.00196 24904430 PMC4034414

[71] Mallat Z Corbaz A Scoazec A Graber P Alouani S Esposito B. Interleukin-18/Interleukin-18 Binding Protein Signaling Modulates Atherosclerotic Lesion Development and Stability. Circ Res (2001) 89(7):E41–5. doi: 10.1161/hh1901.098735 11577031

[72] Uyemura K Demer LL Castle SC Jullien D Berliner JA Gately MK. Cross-Regulatory Roles of Interleukin (IL)-12 and IL-10 in Atherosclerosis. J Clin Invest (1996) 97(9):2130–8. doi: 10.1172/JCI118650 PMC507288 8621803

[73] Chistiakov DA Kashirskikh DA Khotina VA Grechko AV Orekhov AN. Immune-Inflammatory Responses in Atherosclerosis: The Role of Myeloid Cells. J Clin Med (2019) 8(11). doi: 10.3390/jcm8111798 PMC6912749 31717832

[74] Bonaccorsi I De Pasquale C Campana S Barberi C Cavaliere R Benedetto F. Natural Killer Cells in the Innate Immunity Network of Atherosclerosis. Immunol Lett (2015) 168(1):51–7. doi: 10.1016/j.imlet.2015.09.006 26384623

[75] Raulet DH Gasser S Gowen BG Deng W Jung H. Regulation of Ligands for the NKG2D Activating Receptor. Annu Rev Immunol (2013) 31:413–41. doi: 10.1146/annurev-immunol-032712-095951 PMC4244079 23298206

[76] Bonaccorsi I Spinelli D Cantoni C Barilla C Pipito N De Pasquale C. Symptomatic Carotid Atherosclerotic Plaques Are Associated With Increased Infiltration of Natural Killer (NK) Cells and Higher Serum Levels of NK Activating Receptor Ligands. Front Immunol (2019) 10:1503. doi: 10.3389/fimmu.2019.01503 31354703 PMC6639781

[77] Engelbertsen D Autio A Verwilligen RAF Depuydt MAC Newton G Rattik S. Increased Lymphocyte Activation and Atherosclerosis in CD47-Deficient Mice. Sci Rep (2019) 9(1):10608. doi: 10.1038/s41598-019-46942-x 31337788 PMC6650435

[78] Kojima Y Volkmer JP McKenna K Civelek M Lusis AJ Miller CL. CD47-Blocking Antibodies Restore Phagocytosis and Prevent Atherosclerosis. Nature (2016) 536(7614):86–90. doi: 10.1038/nature18935 27437576 PMC4980260

[79] Solanki A Bhatt LK Johnston TP. Evolving Targets for the Treatment of Atherosclerosis. Pharmacol Ther (2018) 187:1–12. doi: 10.1016/j.pharmthera.2018.02.002 29414673

[80] Dahou S Smahi MC Nouari W Dahmani Z Benmansour S Ysmail-Dahlouk L. L-Threoascorbic Acid Treatment Promotes S. Aureus-Infected Primary Human Endothelial Cells Survival and Function, as Well as Intracellular Bacterial Killing, and Immunomodulates the Release of IL-1beta and Soluble ICAM-1. Int Immunopharmacol (2021) 95:107476. doi: 10.1016/j.intimp.2021.107476 33676147

[81] Leentjens J Bekkering S Joosten LAB Netea MG Burgner DP Riksen NP. Trained Innate Immunity as a Novel Mechanism Linking Infection and the Development of Atherosclerosis. Circ Res (2018) 122(5):664–9. doi: 10.1161/CIRCRESAHA.117.312465 29367213

[82] Pober JS Sessa WC. Evolving Functions of Endothelial Cells in Inflammation. Nat Rev Immunol (2007) 7(10):803–15. doi: 10.1038/nri2171 17893694

[83] Mai J Virtue A Shen J Wang H Yang XF. An Evolving New Paradigm: Endothelial Cells–Conditional Innate Immune Cells. J Hematol Oncol (2013) 6:61. doi: 10.1186/1756-8722-6-61 23965413 PMC3765446

[84] Christ A Bekkering S Latz E Riksen NP. Long-Term Activation of the Innate Immune System in Atherosclerosis. Semin Immunol (2016) 28(4):384–93. doi: 10.1016/j.smim.2016.04.004 27113267

[85] Bekkering S Joosten LA van der Meer JW Netea MG Riksen NP. Trained Innate Immunity and Atherosclerosis. Curr Opin Lipidol (2013) 24(6):487–92. doi: 10.1097/MOL.0000000000000023 24184939

[86] van Tuijl J Joosten LAB Netea MG Bekkering S Riksen NP. Immunometabolism Orchestrates Training of Innate Immunity in Atherosclerosis. Cardiovasc Res (2019) 115(9):1416–24. doi: 10.1093/cvr/cvz107 PMC6910162 31050710

[87] Zmyslowski A Szterk A. Current Knowledge on the Mechanism of Atherosclerosis and Pro-Atherosclerotic Properties of Oxysterols. Lipids Health Dis (2017) 16(1):188. doi: 10.1186/s12944-017-0579-2 28969682 PMC5625595

[88] Choi C Finlay DK. Diverse Immunoregulatory Roles of Oxysterols-The Oxidized Cholesterol Metabolites. Metabolites (2020) 10(10). doi: 10.3390/metabo10100384 PMC7601797 32998240

[89] Sharma BR Kanneganti TD. NLRP3 Inflammasome in Cancer and Metabolic Diseases. Nat Immunol (2021) 22(5):550–9. doi: 10.1038/s41590-021-00886-5 PMC8132572 33707781

[90] Duewell P Kono H Rayner KJ Sirois CM Vladimer G Bauernfeind FG. NLRP3 Inflammasomes are Required for Atherogenesis and Activated by Cholesterol Crystals. Nature (2010) 464(7293):1357–61. doi: 10.1038/nature08938 PMC2946640 20428172

[91] Dupaul-Chicoine J Arabzadeh A Dagenais M Douglas T Champagne C Morizot A. The Nlrp3 Inflammasome Suppresses Colorectal Cancer Metastatic Growth in the Liver by Promoting Natural Killer Cell Tumoricidal Activity. Immunity (2015) 43(4):751–63. doi: 10.1016/j.immuni.2015.08.013 26384545

[92] Lee HH Kim D Jung J Kang H Cho H. NLRP3 Deficiency in Hepatocellular Carcinoma Enhances Surveillance of NK-92 Through a Modulation of MICA/B. Int J Mol Sci (2021) 22(17). doi: 10.3390/ijms22179285 PMC8430511 34502191

[93] Liu G Atteridge CL Wang X Lundgren AD Wu JD. The Membrane Type Matrix Metalloproteinase MMP14 Mediates Constitutive Shedding of MHC Class I Chain-Related Molecule A Independent of A Disintegrin and Metalloproteinases. J Immunol (2010) 184(7):3346–50. doi: 10.4049/jimmunol.0903789 PMC3191873 20208009

[94] Xia M Guerra N Sukhova GK Yang K Miller CK Shi GP. Immune Activation Resulting From NKG2D/ligand Interaction Promotes Atherosclerosis. Circulation (2011) 124(25):2933–43. doi: 10.1161/CIRCULATIONAHA.111.034850 PMC3289255 22104546

[95] Laakso M. Hyperglycemia and Cardiovascular Disease in Type 2 Diabetes. Diabetes (1999) 48(5):937–42. doi: 10.2337/diabetes.48.5.937 10331395

[96] Berbudi A Rahmadika N Tjahjadi AI Ruslami R. Type 2 Diabetes and Its Impact on the Immune System. Curr Diabetes Rev (2020) 16(5):442–9. doi: 10.2174/1573399815666191024085838 PMC7475801 31657690

[97] Elksnis A Martinell M Eriksson O Espes D. Heterogeneity of Metabolic Defects in Type 2 Diabetes and Its Relation to Reactive Oxygen Species and Alterations in Beta-Cell Mass. Front Physiol (2019) 10:107. doi: 10.3389/fphys.2019.00107 30837889 PMC6383038

[98] Gerber PA Rutter GA. The Role of Oxidative Stress and Hypoxia in Pancreatic Beta-Cell Dysfunction in Diabetes Mellitus. Antioxid Redox Signal (2017) 26(10):501–18. doi: 10.1089/ars.2016.6755 PMC5372767 27225690

[99] De Lerma Barbaro A Palano MT Cucchiara M Gallazzi M Mortara L Bruno A. Metabolic Rewiring in the Tumor Microenvironment to Support Immunotherapy: A Focus on Neutrophils, Polymorphonuclear Myeloid-Derived Suppressor Cells and Natural Killer Cells. Vaccines (Basel) (2021) 9(10). doi: 10.3390/vaccines9101178 PMC8539473 34696286

[100] Vitale M Parodi M. Blocking HIF to Enhance NK Cells: Hints for New Anti-Tumor Therapeutic Strategies? Vaccines (Basel) (2021) 9(10). doi: 10.3390/vaccines9101144 PMC8539190 34696251

[101] Luo L Lu J Wei L Long D Guo JY Shan J. The Role of HIF-1 in Up-Regulating MICA Expression on Human Renal Proximal Tubular Epithelial Cells During Hypoxia/Reoxygenation. BMC Cell Biol (2010) 11:91. doi: 10.1186/1471-2121-11-91 21092233 PMC3000391

[102] Wei L Lu J Feng L Long D Shan J Li S. HIF-1alpha Accumulation Upregulates MICA and MICB Expression on Human Cardiomyocytes and Enhances NK Cell Cytotoxicity During Hypoxia-Reoxygenation. Life Sci (2010) 87(3-4):111–9. doi: 10.1016/j.lfs.2010.05.012 20566410

[103] Revelo XS Luck H Winer S Winer DA. Morphological and Inflammatory Changes in Visceral Adipose Tissue During Obesity. Endocr Pathol (2014) 25(1):93–101. doi: 10.1007/s12022-013-9288-1 24356782

[104] Newman JD Vani AK Aleman JO Weintraub HS Berger JS Schwartzbard AZ. The Changing Landscape of Diabetes Therapy for Cardiovascular Risk Reduction: JACC State-Of-The-Art Review. J Am Coll Cardiol (2018) 72(15):1856–69. doi: 10.1016/j.jacc.2018.07.071 PMC6178226 30286929

[105] Choudhury RP Edgar L Ryden M Fisher EA. Diabetes and Metabolic Drivers of Trained Immunity: New Therapeutic Targets Beyond Glucose. Arterioscler Thromb Vasc Biol (2021) 41(4):1284–90. doi: 10.1161/ATVBAHA.120.314211 PMC10069665 33657881

[106] Moreno PR Murcia AM Palacios IF Leon MN Bernardi VH Fuster V. Coronary Composition and Macrophage Infiltration in Atherectomy Specimens From Patients With Diabetes Mellitus. Circulation (2000) 102(18):2180–4. doi: 10.1161/01.cir.102.18.2180 11056089

[107] Flynn MC Kraakman MJ Tikellis C Lee MKS Hanssen NMJ Kammoun HL. Transient Intermittent Hyperglycemia Accelerates Atherosclerosis by Promoting Myelopoiesis. Circ Res (2020) 127(7):877–92. doi: 10.1161/CIRCRESAHA.120.316653 PMC7486277 32564710

[108] Barrett TJ Murphy AJ Goldberg IJ Fisher EA. Diabetes-Mediated Myelopoiesis and the Relationship to Cardiovascular Risk. Ann N Y Acad Sci (2017) 1402(1):31–42. doi: 10.1111/nyas.13462 28926114 PMC5659728

[109] Spinetti G Cordella D Fortunato O Sangalli E Losa S Gotti A. Global Remodeling of the Vascular Stem Cell Niche in Bone Marrow of Diabetic Patients: Implication of the microRNA-155/FOXO3a Signaling Pathway. Circ Res (2013) 112(3):510–22. doi: 10.1161/CIRCRESAHA.112.300598 PMC3616365 23250986

[110] Ferland-McCollough D Maselli D Spinetti G Sambataro M Sullivan N Blom A. MCP-1 Feedback Loop Between Adipocytes and Mesenchymal Stromal Cells Causes Fat Accumulation and Contributes to Hematopoietic Stem Cell Rarefaction in the Bone Marrow of Patients With Diabetes. Diabetes (2018) 67(7):1380–94. doi: 10.2337/db18-0044 29703845

[111] Dang Z Maselli D Spinetti G Sangalli E Carnelli F Rosa F. Sensory Neuropathy Hampers Nociception-Mediated Bone Marrow Stem Cell Release in Mice and Patients With Diabetes. Diabetologia (2015) 58(11):2653–62. doi: 10.1007/s00125-015-3735-0 PMC4589553 26358583

[112] Amadesi S Reni C Katare R Meloni M Oikawa A Beltrami AP. Role for Substance P-Based Nociceptive Signaling in Progenitor Cell Activation and Angiogenesis During Ischemia in Mice and in Human Subjects. Circulation (2012) 125(14):1774–86, S1-19. doi: 10.1161/CIRCULATIONAHA.111.089763 22392530 PMC3616366

[113] Santopaolo M Sambataro M Spinetti G Madeddu P. Bone Marrow as a Target and Accomplice of Vascular Complications in Diabetes. Diabetes Metab Res Rev (2019) e3240. doi: 10.1002/dmrr.3240 31840418

[114] Santopaolo M Sullivan N Thomas AC Alvino VV Nicholson LB Gu Y. Activation of Bone Marrow Adaptive Immunity in Type 2 Diabetes: Rescue by Co-Stimulation Modulator Abatacept. Front Immunol (2021) 12:609406. doi: 10.3389/fimmu.2021.609406 33746953 PMC7969721

[115] Mxinwa V Dludla PV Nyambuya TM Mokgalaboni K Mazibuko-Mbeje SE Nkambule BB. Natural Killer Cell Levels in Adults Living With Type 2 Diabetes: A Systematic Review and Meta-Analysis of Clinical Studies. BMC Immunol (2020) 21(1):51. doi: 10.1186/s12865-020-00378-5 32907543 PMC7487809

[116] Francisco CO Catai AM Moura-Tonello SC Arruda LC Lopes SL Benze BG. Cytokine Profile and Lymphocyte Subsets in Type 2 Diabetes. Braz J Med Biol Res (2016) 49(4):e5062. doi: 10.1590/1414-431X20155062 27007651 PMC4819407

[117] Berrou J Fougeray S Venot M Chardiny V Gautier JF Dulphy N. Natural Killer Cell Function, an Important Target for Infection and Tumor Protection, is Impaired in Type 2 Diabetes. PloS One (2013) 8(4):e62418. doi: 10.1371/journal.pone.0062418 23638076 PMC3636194

[118] Piatkiewicz P Milek T Bernat-Karpinska M Ohams M Czech A Ciostek P. The Dysfunction of NK Cells in Patients With Type 2 Diabetes and Colon Cancer. Arch Immunol Ther Exp (Warsz) (2013) 61(3):245–53. doi: 10.1007/s00005-013-0222-5 23456207

[119] Kim JH Park K Lee SB Kang S Park JS Ahn CW. Relationship Between Natural Killer Cell Activity and Glucose Control in Patients With Type 2 Diabetes and Prediabetes. J Diabetes Investig (2019) 10(5):1223–8. doi: 10.1111/jdi.13002 PMC6717814 30618112

[120] Simar D Versteyhe S Donkin I Liu J Hesson L Nylander V. DNA Methylation Is Altered in B and NK Lymphocytes in Obese and Type 2 Diabetic Human. Metabolism (2014) 63(9):1188–97. doi: 10.1016/j.metabol.2014.05.014 24996265

[121] Lee BC Kim MS Pae M Yamamoto Y Eberle D Shimada T. Adipose Natural Killer Cells Regulate Adipose Tissue Macrophages to Promote Insulin Resistance in Obesity. Cell Metab (2016) 23(4):685–98. doi: 10.1016/j.cmet.2016.03.002 PMC4833527 27050305

[122] Kahn SE Hull RL Utzschneider KM. Mechanisms Linking Obesity to Insulin Resistance and Type 2 Diabetes. Nature (2006) 444(7121):840–6. doi: 10.1038/nature05482 17167471

[123] Park J Morley TS Kim M Clegg DJ Scherer PE. Obesity and Cancer–Mechanisms Underlying Tumour Progression and Recurrence. Nat Rev Endocrinol (2014) 10(8):455–65. doi: 10.1038/nrendo.2014.94 PMC4374431 24935119

[124] Shoelson SE Herrero L Naaz A. Obesity, Inflammation, and Insulin Resistance. Gastroenterology (2007) 132(6):2169–80. doi: 10.1053/j.gastro.2007.03.059 17498510

[125] Van Gaal LF Mertens IL De Block CE. Mechanisms Linking Obesity With Cardiovascular Disease. Nature (2006) 444(7121):875–80. doi: 10.1038/nature05487 17167476

[126] Caer C Rouault C Le Roy T Poitou C Aron-Wisnewsky J Torcivia A. Immune Cell-Derived Cytokines Contribute to Obesity-Related Inflammation, Fibrogenesis and Metabolic Deregulation in Human Adipose Tissue. Sci Rep (2017) 7(1):3000. doi: 10.1038/s41598-017-02660-w 28592801 PMC5462798

[127] Febbraio MA. Role of Interleukins in Obesity: Implications for Metabolic Disease. Trends Endocrinol Metab (2014) 25(6):312–9. doi: 10.1016/j.tem.2014.02.004 24698032

[128] Rocha VZ Libby P. Obesity, Inflammation, and Atherosclerosis. Nat Rev Cardiol (2009) 6(6):399–409. doi: 10.1038/nrcardio.2009.55 19399028

[129] Bahr I Goritz V Doberstein H Hiller GG Rosenstock P Jahn J. Diet-Induced Obesity Is Associated With an Impaired NK Cell Function and an Increased Colon Cancer Incidence. J Nutr Metab (2017) 2017:4297025. doi: 10.1155/2017/4297025 28357137 PMC5357539

[130] Bahr I Spielmann J Quandt D Kielstein H. Obesity-Associated Alterations of Natural Killer Cells and Immunosurveillance of Cancer. Front Immunol (2020) 11:245. doi: 10.3389/fimmu.2020.00245 32231659 PMC7082404

[131] Viel S Besson L Charrier E Marcais A Disse E Bienvenu J. Alteration of Natural Killer Cell Phenotype and Function in Obese Individuals. Clin Immunol (2017) 177:12–7. doi: 10.1016/j.clim.2016.01.007 26794911

[132] Viel S Besson L Marotel M Walzer T Marcais A. Regulation of mTOR, Metabolic Fitness, and Effector Functions by Cytokines in Natural Killer Cells. Cancers (Basel) (2017) 9(10). doi: 10.3390/cancers9100132 PMC5664071 28956813

[133] Tobin LM Mavinkurve M Carolan E Kinlen D O'Brien EC Little MA. NK Cells in Childhood Obesity are Activated, Metabolically Stressed, and Functionally Deficient. JCI Insight (2017) 2(24). doi: 10.1172/jci.insight.94939 PMC5752310 29263296

[134] Mauer J Chaurasia B Goldau J Vogt MC Ruud J Nguyen KD. Signaling by IL-6 Promotes Alternative Activation of Macrophages to Limit Endotoxemia and Obesity-Associated Resistance to Insulin. Nat Immunol (2014) 15(5):423–30. doi: 10.1038/ni.2865 PMC4161471 24681566

[135] Bottger E Grangeiro de Carvalho E Meese S Kun JF Esen M. Expression of Interleukin-6 Family Receptors in NK92 Cells is Regulated by Cytokines and Not Through Direct Interaction With Plasmodium Falciparum-Infected Erythrocytes. J Interferon Cytokine Res (2013) 33(2):65–71. doi: 10.1089/jir.2012.0094 23398366

[136] Oberg HH Wesch D Grussel S Rose-John S Kabelitz D. Differential Expression of CD126 and CD130 Mediates Different STAT-3 Phosphorylation in CD4+CD25- and CD25high Regulatory T Cells. Int Immunol (2006) 18(4):555–63. doi: 10.1093/intimm/dxh396 16540526

[137] Theurich S Tsaousidou E Hanssen R Lempradl AM Mauer J Timper K. IL-6/Stat3-Dependent Induction of a Distinct, Obesity-Associated NK Cell Subpopulation Deteriorates Energy and Glucose Homeostasis. Cell Metab (2017) 26(1):171–84.e6. doi: 10.1016/j.cmet.2017.05.018 28683285

[138] Passos ST Silver JS O'Hara AC Sehy D Stumhofer JS Hunter CA. IL-6 Promotes NK Cell Production of IL-17 During Toxoplasmosis. J Immunol (2010) 184(4):1776–83. doi: 10.4049/jimmunol.0901843 PMC3757499 20083665

[139] Pedersen L Idorn M Olofsson GH Lauenborg B Nookaew I Hansen RH. Voluntary Running Suppresses Tumor Growth Through Epinephrine- and IL-6-Dependent NK Cell Mobilization and Redistribution. Cell Metab (2016) 23(3):554–62. doi: 10.1016/j.cmet.2016.01.011 26895752

[140] Champsaur M Lanier LL. Effect of NKG2D Ligand Expression on Host Immune Responses. Immunol Rev (2010) 235(1):267–85. doi: 10.1111/j.0105-2896.2010.00893.x PMC2885032 20536569

[141] Shah NR Braverman ER. Measuring Adiposity in Patients: The Utility of Body Mass Index (BMI), Percent Body Fat, and Leptin. PloS One (2012) 7(4):e33308. doi: 10.1371/journal.pone.0033308 22485140 PMC3317663

[142] Marcais A Cherfils-Vicini J Viant C Degouve S Viel S Fenis A. The Metabolic Checkpoint Kinase mTOR is Essential for IL-15 Signaling During the Development and Activation of NK Cells. Nat Immunol (2014) 15(8):749–57. doi: 10.1038/ni.2936 PMC4110708 24973821

[143] Marcais A Marotel M Degouve S Koenig A Fauteux-Daniel S Drouillard A. High mTOR Activity Is a Hallmark of Reactive Natural Killer Cells and Amplifies Early Signaling Through Activating Receptors. Elife (2017) 6. doi: 10.7554/eLife.26423 PMC5628014 28875936

[144] Starling S. New Therapeutic Promise for Leptin. Nat Rev Endocrinol (2019) 15(11):625. doi: 10.1038/s41574-019-0265-8 31541201

[145] Stern JH Rutkowski JM Scherer PE. Adiponectin, Leptin, and Fatty Acids in the Maintenance of Metabolic Homeostasis Through Adipose Tissue Crosstalk. Cell Metab (2016) 23(5):770–84. doi: 10.1016/j.cmet.2016.04.011 PMC4864949 27166942

[146] Barberio AM Alareeki A Viner B Pader J Vena JE Arora P. Central Body Fatness is a Stronger Predictor of Cancer Risk Than Overall Body Size. Nat Commun (2019) 10(1):383. doi: 10.1038/s41467-018-08159-w 30670692 PMC6342989

[147] Quail DF Dannenberg AJ. The Obese Adipose Tissue Microenvironment in Cancer Development and Progression. Nat Rev Endocrinol (2019) 15(3):139–54. doi: 10.1038/s41574-018-0126-x PMC6374176 30459447

[148] Khafagy R Dash S. Obesity and Cardiovascular Disease: The Emerging Role of Inflammation. Front Cardiovasc Med (2021) 8:768119. doi: 10.3389/fcvm.2021.768119 34760952 PMC8573144

